# New Parvoviruses and Picornavirus in Tissues and Feces of Foals with Interstitial Pneumonia

**DOI:** 10.3390/v13081612

**Published:** 2021-08-14

**Authors:** Eda Altan, Alvin Hui, Yanpeng Li, Patricia Pesavento, Javier Asín, Beate Crossley, Xutao Deng, Francisco A. Uzal, Eric Delwart

**Affiliations:** 1 Vitalant Research Institute, San Francisco, CA 94118, USA; edaltan@hotmail.com (E.A.); ahui@vitalant.org (A.H.); alphaleeyp@hotmail.com (Y.L.); xdeng@vitalant.org (X.D.); 2 Department of Laboratory Medicine, University of California at San Francisco, San Francisco, CA 94118, USA; 3 Department of Pathology Microbiology and Immunology, UC Davis, Davis, CA 95616, USA; papesavento@ucdavis.edu (P.P.); jasinros@ucdavis.edu (J.A.); fauzal@ucdavis.edu (F.A.U.); 4 California Animal Health and Food Safety Laboratory System, UC Davis, Davis, CA 95616, USA; bcrossley@ucdavis.edu; 5 Department of Medicine and Epidemiology, UC Davis, Davis, CA 95616, USA

**Keywords:** *Equus caballus*, cabavirus, metagenomics, foal, parvovirus, picornavirus

## Abstract

Six foals with interstitial pneumonia of undetermined etiology from Southern California were analyzed by viral metagenomics. Spleen, lung, and colon content samples obtained during necropsy from each animal were pooled, and nucleic acids from virus-like particles enriched for deep sequencing. The recently described equine copiparvovirus named eqcopivirus, as well as three previously uncharacterized viruses, were identified. The complete ORFs genomes of two closely related protoparvoviruses, and of a bocaparvovirus, plus the partial genome of a picornavirus were assembled. The parvoviruses were classified as members of new ungulate protoparvovirus and bocaparvovirus species in the *Parvoviridae* family. The picornavirus was classified as a new species in the *Salivirus* genus of the *Picornaviridae* family. Spleen, lung, and colon content samples from each foal were then tested for these viral genomes by nested PCR and RT-PCR. When present, parvoviruses were detected in both feces and spleen. The picornavirus, protoparvovirus, and eqcopivirus genomes were detected in the lungs of one animal each. Three foals were co-infected with the picornavirus and either a protoparvovirus, bocaparvovirus, or eqcopivirus. Two other foals were infected with a protoparvovirus only. No viral infection was detected in one animal. The complete ORFs of the first equine protoparvoviruses and bocaparvovirus, the partial ORF of the third equine picornavirus, and their detection in tissues of foals with interstitial pneumonia are described here. Testing the involvement of these viruses in fatal interstitial pneumonia or other equine diseases will require larger epidemiological and/or inoculation studies.

## 1. Introduction

Over the last century, numerous equine viruses have been isolated and described [1,2,3,4] including equid alphaherpesviruses one and four (EHV-1 and EHV-4), associated with respiratory and neurological disease [5,6,7]. There are multiple reports of sporadic acute interstitial pneumonia in foals of undetermined etiology [8]. Viral and toxic causes have been proposed, but not definitely proved [9,10]. This condition has been recognized in California over the years [11] and has been given multiple names (e.g., acute interstitial pneumonia of foals, severe bronchointerstitial pneumonia of foals, foal acute respiratory distress syndrome, or foal interstitial pneumonia) [8,9,10,11,12]. To date no metagenomics studies to investigate equine interstitial pneumonia’s possible viral etiology have been reported.

Metagenomics characterizations of the equine virome have revealed the presence of previously unknown eukaryotic viruses. One of the earliest mammalian viral metagenomics studies, published in 2005, analyzed horse feces and identified mainly phages [13]. Small circular replication-associated protein (Rep)-encoding single-stranded DNA genomes (CRESS) of unknown tropism called gemycircularvirus were recently characterized in horse feces [14]. The alphavirus Getah virus, typically infecting pigs, was detected in the plasma of febrile horses in China using viral metagenomics [15]. A flavivirus in the pegivirus genus named Theiler’s disease-associated virus (TDAV) was characterized using meta-transcriptomics and was initially thought to cause equine serum hepatitis [16]. Another pegivirus was detected by consensus PCR in horse plasma [17]. A flavivirus in the hepacivirus genus, initially identified in dog plasma, was subsequently shown to also be a common but asymptomatic infection in horses [18]. None of these 3 RNA viruses was reproducibly associated with equine serum hepatitis [19,20]. More recently, a copiparvovirus named equine parvovirus-H (EqPV-H) was characterized through plasma deep sequencing. Although many parvovirus infections are asymptomatic, EqPV-H was shown to be associated with, and able to induce, hepatitis following inoculation [21,22]. A study of sterile and non-sterile (e.g., feces) samples from horses also revealed the presence of several circular DNA viral genomes of unknown tropism, as well as a second equine copiparvovirus named EqPV-CSF, in a horse with neurological problems [23]. More recently, a metagenomics study of plasma and CSF from horses with respiratory and neurological problems revealed the genome of a third equine copiparvovirus named eqcopivirus [24]. Testing a limited number of horses, none of the three currently known equine copiparvoviruses (EqPV-H, EqPV-CSF, and eqcopivirus) could be associated with either neurological or respiratory problems [24]. Circovirus DNA was also recently identified by viral metagenomics in the plasma of a horse with hepatitis [25].

Foal interstitial pneumonia has been observed sporadically in California over the years, especially in spring and summer months [11]. It usually affects individual foals within premises, but small clusters have also been reported. Interstitial or bronchointerstitial pneumonia, with occasional hyaline membranes, and different degree of proliferative response and chronicity, has been detected [9,10]. Sporadic isolation of *Rhodococcus equi* has been reported in some cases [8], but in general no significant bacteria is consistently cultured. Despite the clinicopathologic resemblance to certain respiratory viral infections, no association with EHV-1, EHV-4, or equine influenza virus has been made to date. 

Here, we analyzed lung, spleen, and feces from six horses from three ranches with unexplained fatal interstitial pneumonia, compatible with previous descriptions of foal interstitial pneumonia and reports on the detection of eqcopivirus as well as the characterization of four previously unknown equine viral genomes: two closely related protoparvoviruses, one bocaparvovirus, and a picornavirus.

## 2. Materials and Methods

### 2.1. Animal Samples Collection and Diagnostic Testing

Samples were collected from dead horses submitted for post-mortem examination and diagnostic work up to the California Animal Health and Food Safety Laboratory, UC Davis, with owners’ consent. UC Davis does not require or provide an IACUC protocol to work with samples collected from dead animals submitted for diagnostic purposes. The gender, age, and breed of studied foals are listed in Table 1.

Full necropsies were performed on all the foals. Multiple ancillary tests were performed following CAHFS standard operating procedures. Briefly, formalin-fixed lung tissue samples were routinely processed for the production of 4 µm hematoxylin and eosin-stained sections that were examined with a light microscope. Lung swabs were inoculated onto Columbia 5% and chocolate agar plates (Hardy Diagnostics, Santa Maria, CA, USA), which were incubated at 37 °C for 48 h under aerobic conditions. 

Virus isolation was attempted using Madin–Darby bovine kidney cells (MDBK) (ATCC, Manassas, VA, USA), Madin–Darby canine kidney cells (MDCK) (ATCC, Manassas, VA, USA), and baby hamster kidney cells (BHK-13) (ATCC, Manassas, VA, USA) supplemented with 2% fetal bovine serum (FBS) (SAFC, MilliporeSigma, St. Louis, MO, US) in minimum essential media (MEM)(Corning, Mediatech Inc, Manassas, VA, USA). Prior to homogenizing the sample using M-tubes with gentleMACS Octo dissociator (Miltenyi Biotec, Bergisch Gladbach, Germany), a 10% tissue suspension was prepared by macerating lung tissue with a scalpel and adding 2 × DD (2 times diagnostic diluent consisting of MEM with penicillin streptomycin (Invitrogen, Thermo Fisher, Waltham, MA, USA), ciprofloxacin (MP Biomedicals, Santa Ana, CA, USA), and amphotericin B (Sigma, St. Louis, MO, USA). The swab samples were vigorously agitated, and the swab fluid was diluted by adding 2 × DD to the final volume, then centrifuged at 1800× *g* at 4 °C for 20 min. The resulting supernatant was inoculated onto 70–90% confluent cells, then incubated at 37 °C for one hour. The inoculum was removed, and the monolayer was rinsed once using 2% FBS/MEM maintenance media. The cell cultures were fed (2% Fetal Bovine Serum/MEM) and observed for cytopathic effects daily for 7 days. Two cell culture passages were evaluated. 

Additionally, 50 ul of swab fluid was extracted (Max96Viral RNA Isolation kit [AM 1836-5], Life Technologies, Carlsbad, CA, USA) to prepare samples for real-time PCR testing of equid influenza, EHV-1, and EHV-4, following the recommendations of the manufacturer. Lung tissue samples were homogenized using a MagNA Lyser (Roche Diagnostics, Basel, Switzerland). Briefly, 250 mg of tissue was placed in a 2 mL polypropylene tube containing 1.5 mL of denaturation solution #8540G (Ambion Thermo Fisher, Waltham, MA, USA) and filled a quarter full with silica beads. The sample was homogenized at 6500 rpm for 45 s, then incubated at room temperature for 5 min. Next, 20 µL of Proteinase K (20 mg/mL) was mixed with 20 µL of homogenate and incubated at 56 °C for 60 min. The digest was then transferred to a MagMax 96 Express plate (Thermo Fisher, Waltham, MA, USA) with 20 µL of binding beads and 100 µL lysis solution per sample. The plate was transferred to a King Fisher Flex magnetic processor (Thermo Fisher, Waltham, MA, USA) and RNA extraction was performed using the AM1836 extraction protocol as described in the MagMax-96 Viral RNA Isolation Kit user guide (Thermo Fisher, Waltham, MA, USA). 

Real-time PCR for the detection of EHV-1 and EHV-4 was performed using the Qiagen QuantiTect Multiplex PCR Mastermix kit (Qiagen, Hilden, Germany). Samples were tested using a multiplex PCR capable of distinguishing between EHV-1 and EHV-4 strains [26,27] and a national animal health laboratory network (NAHLN) real-time PCR for influenza virus matrix gene [28] on an Applied Biosystems 7500 Fast Real-Time PCR System (Life Technologies, Carlsbad, CA, USA). The PCR used to detect the presence of the equine viruses detected in this study used the following thermocycler conditions: initial denaturation 95 °C for 5 min, denaturation for 30 s at 95 °C, annealing at 57 °C for 30 s, extension at 72 °C for 60 s for 45 cycles, final extension for 10 min, hold at 4 °C. For the first round, bocaparvovirus PCR extension time was 90 s. The PCR primers for the two closely related protoparvoviruses were designed over conserved nucleotide regions. The primers used are shown in Table 2.

### 2.2. Metagenomics

Virus metagenomics library preparation and bioinformatics analyses were performed as described [29,30]. Viral-like particles (VLP) were enriched by homogenization of spleen and lung tissues and colon content from each horse followed by filtration and digestion with nuclease enzymes of supernatants to reduce the concentration of non-viral, capsid-protected, nucleic acids. The resulting enriched VLP from the different samples from each of the foals were then pooled. Following nucleic acids extraction of the six pools and random RT-PCR, the DNA amplification products were converted to Illumina compatible DNA using a Nextera™ XT Sample Preparation Kit with dual barcoding and analyzed on an Illumina MiSeq using 250 bases paired end sequencing resulting in six initial sequence datasets (Illumina, Inc., San Diego, CA, USA). A HiSeq (Illumina, Inc., San Diego, CA, USA), run using 150 bases paired end sequencing, was also performed on a subset of individual tissue and colon content samples in order to increase genome coverage. These readings were submitted to GenBank under Bioproject accession number PRJNA724703. 

### 2.3. Bioinformatics

Illumina sequence data analyses were conducted as described using BLASTx to detect reads and contigs showing translated protein sequences’ similarity to all know eukaryotic viruses in RefSeq of GenBank. An in-house pipeline was used for removing bacterial and host genomes, trimming adaptor and primers, and de novo read assembly [29,30]. BLASTx was used for translated protein sequence similarity searches to all viral protein sequences in the GenBank database. To map readings to reference viral genome contigs and generate complete genomes, design PCR primers to bridge genome gaps, and to align multiple genomes and proteins the Geneious R10 program was used (Biomatters Ltd., Auckland, New Zealand). MEGAX software (Pennsylvania State University, PA, USA) used for the creation of phylogenetic trees. Potential splice donor (SD) and acceptor (SA) sites were predicted using the NetGene2 Server (DTU Bioinformatics, Technical University of Denmark) tool [31].

### 2.4. Phylogenetics

The amino acid (aa) pairwise alignments of parvoviruses non-structural protein 1 (NS1) and capsid protein (VP1) and picornaviruses partial 3CD (186aa) were performed using the Geneious R10 program (Biomatters Ltd., Auckland, New Zealand) with the in-built MAFFT algorithm. The amino acid phylogenetic trees were constructed using the maximum likelihood method with two substitution models: the Le Gascuel 2008 model based with gamma distributed (G+) for NS1 and VP1 and invariant sites (G + I) for 3CD in MEGA software version X [32,33]. Bootstrap values (based on 100 replicates) for each node are shown if >70%. 

## 3. Results

Six foals from three ranches in Southern California that died with a history of respiratory disease were submitted to the San Bernardino laboratory of the California Animal Health and Food Safety laboratory system (CAHFS) for necropsy and diagnostic workup. Histology revealed interstitial pneumonia in all the cases, and this lesion was considered to be the cause of death in 5/6 cases (1–2, 4–6) and a significant contributor to clinical signs in case 3. The clinical status of these horses, together with the results of several ancillary diagnostic tests, is shown in Table 3. The bacteriology results were unremarkable. Lung tissue was also tested for EHV-1 and EHV-4 by PCR and equid influenza RNA by RT-PCR (all negative). Lung tissues were also negative for cytopathic effects during virus isolation (VI) attempts on three cells lines. A definitive etiology for the pulmonary lesions could therefore not be identified for these six horses with interstitial pneumonia.

The available tissues for viral metagenomics studies consisted of lung and spleen as well as fecal material collected from the colon content of each foal during necropsies. VLP from tissues and feces from each foal were pooled and six pools sequenced using Illumina technology (Materials and Methods). 

Bioinformatic analysis of the resulting data for the presence of known and novel viral sequences showed the presence of a known equine parvovirus named eqcopivirus, and of divergent sequences indicating the presence of “new” viruses in the *Parvoviridae* and *Picornaviridae* families. De novo assembly was used to assemble two complete ORFs protoparvoviruses and one bocaparvovirus genomes. 

**Figure 1:**
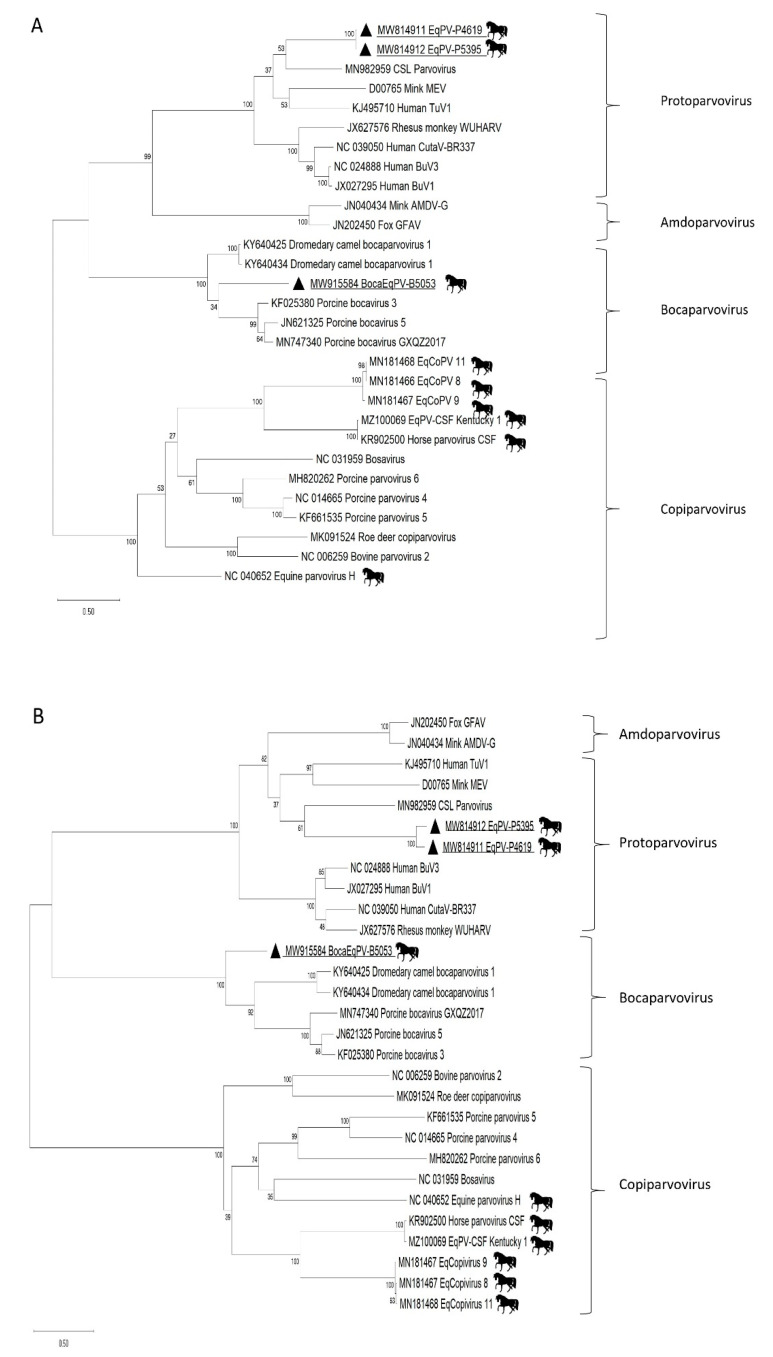
(**A**) Phylogenetic analysis of equine parvoviruses NS1. (**B**) Phylogenetic analysis of equine parvoviruses VP1. The scale indicates amino acid substitutions per position. The amino acid (aa) pairwise alignments were performed with Geneious R10 software using the in-built MAFFT algorithm. The phylogenetic trees were constructed using the maximum likelihood method with substitution model: Le Gascuel 2008 based model with gamma-distributed (G+) for NS1 and VP1 in MEGA software version X. Viral taxa from this study are highlighted with triangles.

The two new protoparvoviruses were 91% similar at the nucleotide level. Their NS1 and VP1 proteins showed 99.3% and 88% identities. The closest relatives of these proteins in GenBank’s non-redundant database (in 1 March 2021) were from a lymph node of a California Sea Lion, protoparvovirus strain Hanchett_2 (MN982959) [29] with amino acid NS1 and VP1 identities of 49% and 40%. For the bocaparvovirus, the closest NS1 and VP1 proteins were from Dromedary camel bocaparvovirus 1 (KY640425) [34] from camels with amino acid identities of 54% and 50%. Phylogenetic analyses for NS1 and VP1 show the relationship of these equine protoparvoviruses and bocaparvovirus proteins to those of other parvoviruses (Figure 1).

**Figure 2:**
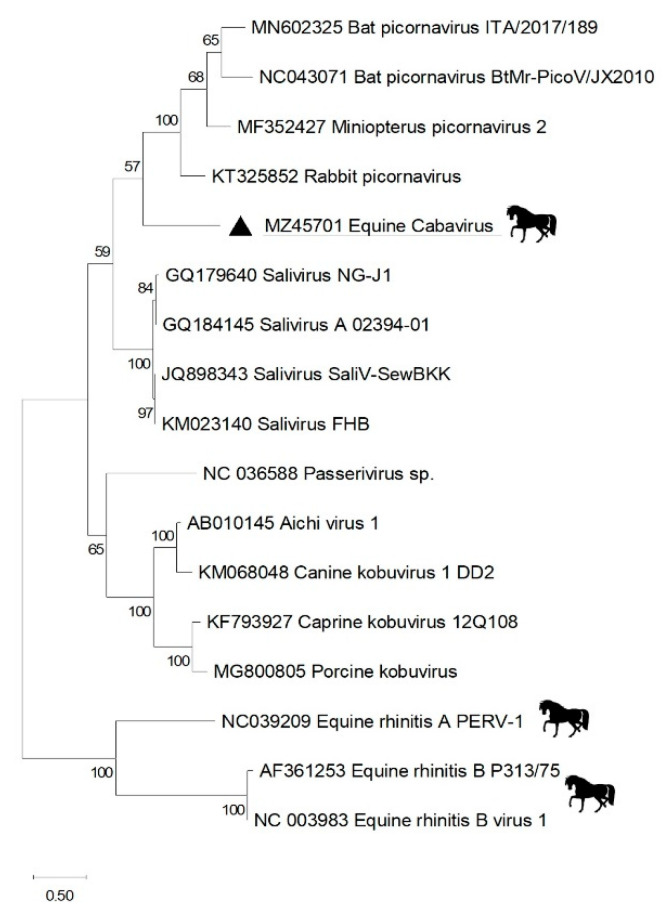
Phylogenetic analysis of RdRp (3CDpol) protein region of novel equine cabavirus picornavirus. The amino acid phylogenetic tree was constructed using the maximum likelihood method with two substitution models: Le Gascuel 2008 model based with gamma distributed, invariant sites (G + I) for 3CD in MEGA software version X. Viral taxon from this study is highlighted with triangle.

Only ~93% of the polyprotein’s ORF of the novel picornavirus could be sequenced. Missing from the final deposited sequences (MZ457016) is the coding sequence for the L protein at the amino terminus end of the P1 region hypothesized to be present based on the closest saliviruses and kobuviruses virus relatives. Also missing was a short nucleotide segment between P1 and P2 region. The closest relatives in the P1 domain (minus likely missing L protein) were from bat picornaviruses from *Minipteros schreibersii* (AWK02686 at 52% identity), *Miniopterus fuliginosus* (AIF74255 at 52%)— from China—and *Pipistrellus pipistrellus* (QIC50030)—from Italy. The closest relatives of the 3Dpol domain were from rabbit picornaviruses (ALO65139 and YP_009513029) with identities of 46–46.5% and human salivirus (ALF96101) with identity of 44.3%. Phylogenetic analysis of the 3CDpol partial region shows the relationship of these RdRp proteins (Figure 2). Based on the ICTV genetic criterion of P1cap and 3Dpol needing identities of less than 34% in P1 and 36% in 3Dpol to qualify as members of a new genus, this picornavirus represents a new species in the *Salivirus* genus. 

The presence of each virus in each of the animal tissues available was then tested using nested PCR for the 3 parvoviruses (eqcopivirus, new closely related protoparvoviruses, and new bocaparvovirus) or RT-nested PCR for the new picornavirus (Table 4). 

One foal was infected with the new bocaparvovirus, one with eqcopivirus, three with the new protoparvoviruses, and three with the new picornavirus. The 253 bases of the protoparvovirus PCR amplicon in the spleen of animal 2 differed from the sequences of animals 1 and 4 by three and two bases, respectively, possibly reflecting the presence of a third variant of that novel ungulate protoparvovirus species. The 305 bases of picornavirus cabavirus amplicons from animals 1, 5, and 6 were identical. Detection of specific viruses was not restricted to particular ranches as the picornavirus was detected on ranch A and C, and the protoparvoviruses on ranch A and B. Three animals were co-infected with picornavirus and either protoparvovirus (animal 1), eqcopivirus (animal 5), or bocaparvovirus (animal 6). Protoparvovirus, eqcopivirus, and cabavirus picornavirus nucleic acids were detected in the lungs of one foal each. 

## 4. Discussion

The equine viruses most commonly associated with respiratory disease are EHV-1, EHV-4, and equine influenza [5,35,36,37,38]. The six foals with fatal interstitial pneumonia analyzed here tested negative for equid influenza viral RNA, and EHV-1 and EHV-4 DNA, and no significant bacteria were isolated (Table 3). Coupled with the clinicopathologic presentation, these cases fulfilled the criteria of the so-called foal interstitial pneumonia (syn. acute interstitial pneumonia of foals, severe bronchointerstitial pneumonia of foals, foal acute respiratory distress syndrome), a syndrome of unidentified etiology that has been reported over the years in California and other parts of the world [8,9,10,11,12].

The metagenomics approach used VLP from pools of lung, spleen, and fecal samples. The near complete genomes of several previously unknown equine viruses were characterized. 

The new picornavirus’ partial genome was sufficiently divergent to be classified as a member of a new novel species that we provisionally called cabavirus in the *Salivirus* genus. Currently known equine picornaviruses consist of equine rhinitis A virus (ERAV) and equine rhinitis B virus (ERBV) in the *Aphthovirus* and *Erbovirus* genera, respectively [39,40,41]. Both are highly prevalent based on serology [42], although their contribution to disease remains uncertain [43]. Cabavirus picornavirus RNA was detected in the fecal sample of two horses; one of which was also positive in the spleen, while a third horse was only nested RT-PCR positive in its lung sample. Whether this picornavirus infection alone or in combination with a protoparvovirus was responsible for its host’s fatal pneumonia is unknown. The most closely related, although still highly divergent, picornaviruses were from bats and rabbits (Figure 2), likely reflecting our still limited but rapidly growing knowledge of picornavirus diversity in mammals [44].

The new protoparvovirus genomes found in the feces and spleens of three horses (including one co-infected with the picornavirus cabavirus) are the first members of this genus to be reported in horses. While two nearly fully sequenced protoparvoviruses were found in foals from the same farm who died only ~2 months apart, the significant level of genetic divergence between their genomes (~9% nucleotide difference) indicates that they diverged long prior to the infection of either horse. The nested PCR detection of a third member of that protoparvovirus species with a few mutations in a short amplicon relative to the other two genomes also reflects a high level of genetic diversity in this viral species. Protoparvovirus DNA was detected in feces, spleen, and lung. The closest relative in the NS1 loci was from a California sea lion parvovirus and in the VP1 loci from a mink parvovirus. The still considerable genetic distance between this equine and other protoparvoviruses likely also reflects our still limited understanding of parvovirus diversity [45,46]. 

A new bocaparvovirus was also detected in the feces and spleen of a single horse. Bocaparvovirus 1 infections in children have been associated with respiratory diseases [47], while other human bocaviruses may cause diarrhea [48,49]. In animals, the bocaviruses named bovine parvovirus and canine minute virus have been associated with diseases of both the respiratory and gastrointestinal track [50]. The bocaparvovirus genome characterized here, the first in a horse, was detected in both the spleen and fecal samples of a single foal. The genetically closest, although also still highly divergent, bocaviruses were from dromedary camel and pigs, which, given their frequent physical proximity to horses, provide potential, although also not recent, common ancestors for this equine bocavirus. 

A single horse was shown to be infected with eqcopivirus, with feces, spleen, and lung all PCR positive (Table 4). Eqcopivirus’ genome was first described in 2019 and detected in the respiratory swabs of 3/13 and in the plasma of 4/14 horses with respiratory problems. Additionally, 7/41 plasma samples from healthy horses were also eqcopivirus DNA positive [24]. The lack of available respiratory swabs from healthy control horses in this prior study precluded testing lung disease association with eqcopivirus detection [24].

Infections with equine hepacivirus, Eq-PV-H, eqcopivirus, or horse parvovirus-CSF are frequently asymptomatic [21,22,24,51,52,53]. The detection of eqcopivirus, protoparvovirus, and picornavirus nucleic acids in the lungs of one foal each supports the possibility but does not prove a role for these viruses in interstitial pneumonia. Future studies to test the potential involvement of these viruses in interstitial pneumonia or other equine diseases could include in situ RNA hybridization to localize viral replication near sites of pathology in lungs or other tissues, epidemiological studies comparing diseased cases and healthy matched controls, and, ultimately, viral inoculations. Whether the foals’ general health, genetic background, passive immunity, or co-infections play supporting roles for possible disease induction by these viruses also remain outstanding questions.

## Figures and Tables

**Table 1:** Characteristics of foals.

ID	Accession	Ranch	Date of Death	Gender	Age	Breed
1	S2002675	A	27 March 2020	Female	3 days	American Quarter Horse
2	S2003716	B	13 May 2020	Male	2 month	American Quarter Horse
3	S2004217	A	5 May 2020	Male	3.5 month	American Quarter Horse
4	S2004619	A	11 June 2020	Female	4 month	American Quarter Horse
5	S2005053	C	28 June 2020	Male	2.5 month	Thoroughbred
6	S2005395	A	7 August 2020	Male	3.5 month	American Quarter Horse

**Table 2:** PCR primers used for detection of four equine viruses.

Virus Target	Nested PCR Primers	Amplicon
**Eqcopivirus**	EqCopiV F1: CAAGGGACCCGAGCCGCCCC	514 bp
	EqCopiV R1: GGGCTGGGGTCTGTGTCCCC	
	EqCopiV F2: GAAAATGTAGAGGTAATTGG	
	EqCopiV R2: GGAATTCCTCAGGGTTTGCC	
**Protoparvoviruses**	EqProtoV F1: TCATCATCATGATCTGGGCC	253 bp
	EqProtoV R1: ACCCAGTTGCCAAATTTGCC	
	EqProtoV F2: TCTATCCGCCAGGTCAGTGG	
	EqProtoV R2: TGATAAAGTTCACGTCTGCC	
**Bocaparvovirus**	EqBocaV F1: GGCCCTTGTTGCACTTGTGG	984 bp
	EqBocaV R1: GCTGCGTTTACAGGCTCCCC	
	EqBocaV F2: CACACTACTTCTCCAGACGG	
	EqBocaV R2: CCTGGAAATAACCACCCTCC	
**Picornavirus**	EqPicoV F1: CTACCCATGGTCGGCTAAGG	306 bp
	EqPicoV R1: TTCGTACGATGCGAAGTCCC	
	EqPicoV F2: GGAGGACCAGACGTTCACTG	
	EqPicoV R2: AGAAGCGAGTCCAGTCCAC	

**Table 3:** Routine analyses results of foals with interstitial pneumonia.

ID	Accession	Dx	Bacteriology	EHV-1 PCR (Lung)	EHV-4 PCR (Lung)	Influenza PCR (Lung)	VI (Lung)	Other Tests and Relevant Findings
**1**	**S2002675**	Int Pneum	Aerobic: mixed flora (liver), no growth (lung) Salmonella culture: Not detected (liver)	NEG	NEG	ND	ND	HMS + Se: Suboptimal Se, 0.21 ppm (Ref range 0.3–1.0); liver
**2**	**S2003716**	Int Pneum	Aerobic: No growth (lung)	NEG	NEG	NEG	ND	HMS + Se: Suboptimal Se, 0.22 ppm (Ref range 0.3–1.0); liver
**3**	**S2004217**	Int Pneum	Aerobic: *E. coli* and Enterococcus hirae (liver), mixed flora (lung), *E. coli* and mixed flora (small intestine), *E. coli* and mixed flora (colon), E. hirae and *E. coli* (abdomen), Staph haemolyticus, mixed flora, *E. coli* (joint) Anaerobic: No growth (small intestine, colon) C. diff culture: Not detected (small intestine, colon) Salmonella culture: Not detected (liver, small intestine, colon)	NEG	NEG	NEG	ND	Fecal float: No parasite eggs/oocysts detected HMS + Se: Acceptable/non-diagnostic ranges; liver Peritonitis with GI content (perforation)
**4**	**S2004619**	Int Pneum	Aerobic: No growth (lung, liver) Salmonella culture: Not detected (liver)	NEG	NEG	NEG	NEG	HMS + Se: Acceptable/non-diagnotic ranges; liver Fecal float: Parascaris eggsSevere ascariasis
**5**	**S2005053**	Int Pneum	Aerobic: No growth (lung, liver) Salmonella culture: Not detected (liver)	NEG	NEG	NEG	ND	Fecal float: No parasite eggs/oocysts detected HMS + Se: Suboptimal Se, 0.23 ppm (Ref range 0.3–1.0); liver
**6**	**S2005395**	Int Pneum	Aerobic: *E. coli* (liver), Acinetobacter Iwoffi and mixed flora (lung) Salmonella culture: Not detected (small intestine)	NEG	ND	NEG	NEG	Fecal float: No parasite eggs/oocysts detected HMS + Se: Acceptable/non-diagnostic ranges; liver

Footnote: VI: Viral isolation; HMS: Heavy metal screen; Se: Selenium; NEG: Negative; ND: Not done.

**Table 4:** nPCR and RT-nPCR detection of three parvoviruses and one picornavirus.

ID	Accession	Ranch	Tissue	Bocavirus	Eqcopivirus	Protoparvovirus	Picornavirus
**1**	**S2005395**	A	Lung	Negative	Negative	Negative	Negative
			Spleen	Negative	Negative	Positive	Positive
			Colon Content	Negative	Negative	Positive	Positive
**2**	**S2002675**	B	Lung	Negative	Negative	Negative	Negative
			Spleen	Negative	Negative	Positive	Negative
**3**	**S2003716**	A	Lung	Negative	Negative	Negative	Negative
			Spleen	Negative	Negative	Negative	Negative
			Colon Content	Negative	Negative	Negative	Negative
**4**	**S2004619**	A	Lung	Negative	Negative	Positive	Negative
			Spleen	Negative	Negative	Positive	Negative
			Colon Content	Negative	Negative	Positive	Negative
**5**	**S2004217**	C	Lung	Negative	Positive	Negative	Negative
			Spleen	Negative	Positive	Negative	Negative
			Colon Content	Negative	Positive	Negative	Positive
**6**	**S2005053**	A	Lung	Negative	Negative	Negative	Positive
			Spleen	Positive	Negative	Negative	Negative
			Colon Content	Positive	Negative	Negative	Negative

Cells in orange indicate first and nested PCR both positive. Cells in yellow indicate only second round nested PCR positive.

## Data Availability

All sequencing data deposited in GenBank under SRA and accession number PRJNA724703 for the Illumina reads and MW814911, MW814912, MW915584, MZ457016 for the virus genomes.
